# Assessment on surface integrity in electrochemical grinding of AISI 304

**DOI:** 10.1016/j.heliyon.2024.e41435

**Published:** 2024-12-24

**Authors:** Mohammad Yazdani, Amir Rasti

**Affiliations:** Advanced Technologies of Machine Tools (ATMT) Lab, Faculty of Mechanical Engineering, Tarbiat Modares University, Tehran, Iran

**Keywords:** Electrochemical grinding, AISI 304, Surface integrity

## Abstract

Electrochemical grinding (ECG) offers advantages such as burr-free and stress-free material removal. Despite its proven potential, limited research has addressed the comprehensive effects of key process parameters on the surface integrity of AISI 304 stainless steel, particularly for applications requiring high-quality finishes, such as medical components. This study bridges this gap by systematically investigating the influence of ECG key parameters including voltage, rotational speed, and electrolyte concentration on main surface integrity parameters including current density, surface roughness, microhardness, and surface texture. Total of 20 experiments were carried out following a Response Surface Methodology (RSM) design, incorporating five levels of variation for the parameters of electrolyte concentration, voltage, and grinding wheel speed. Results revealed that voltage and electrolyte concentration were the dominant factors affecting current density, increasing it by 368 % and 241 %, respectively, while higher rotational speeds decreased it by 44.5 % due to reduced contact time and electrolyte removal. Surface roughness decreased by up to 65 % in the perpendicular direction as concentration and voltage increased, but higher voltages led to over-etching, increased the surface roughness. Electrolyte concentration and voltage also reduced surface microhardness by 10–14 % through intensified corrosion, while higher wheel speeds increased microhardness due to enhanced mechanical removal. The maximum variation for in-depth microhardness extended up to a depth of 40 μm below the surface. Surface texture analysis also revealed more uniform pitting across the surface at higher concentrations, indicating more consistent material dissolution. However, at higher voltages, deep pitting emerged, raising surface roughness.

## 1 Introduction

AISI 304 stainless steel, widely used in various industries for its excellent corrosion resistance, strength, and formability. Among its numerous applications, the manufacturing of medical needles, surgical instruments, and implants is of paramount importance [1,2]. The conventional machining of this alloy often resulting in poor surface quality, increased tool wear, and compromised functionality of the medical devices. However, Medical needles, in particular, demand precision and high surface integrity to ensure minimal tissue damage, reduce patient discomfort, and prevent infection [3,4]. For example, the tip of the needle must be ground to a fine point with a smooth finish, requiring a machining process that can achieve superior surface integrity [5]. Traditional grinding methods can struggle to meet these stringent requirements due to the generation of excessive heat, mechanical stresses, and burr formation.

Electrochemical grinding (ECG) emerges as a highly effective solution to these challenges. ECG combines the principles of electrochemical machining and mechanical grinding, enabling the precise removal of material with minimal thermal and mechanical impact. This hybrid process dissolves more than 80 % of material at the workpiece surface electrochemically, while the grinding wheel continuously removes the remaining material and resultant altered layer mechanically, ensuring a smooth and controlled material removal rate (MRR) [6]. The contact nature of the electrochemical reaction minimizes tool wear and eliminates burr formation, which is crucial for medical applications. Like other cutting processes, ECG involves a set of critical process parameters that must be meticulously optimized to achieve the best results [7]. Key parameters in ECG include the electrolyte composition and concentration, flushing method, voltage, feed rate, and characteristics of the grinding wheel (abrasive grit size, and composition) [8]. These parameters also determine the portion of chemical and mechanical works. The electrolyte, typically a conductive fluid, plays a crucial role in the electrochemical reaction, influencing the MRR and surface finish. Voltage and current density dictate the intensity of the electrochemical dissolution, while the feed rate affects the mechanical grinding action and overall machining efficiency. Fine-tuning these parameters is essential to minimize defects such as surface roughness, thermal damage, and tool wear, thereby ensuring optimal surface integrity and dimensional accuracy of the machined components [9]. For medical applications, achieving a precise balance of these parameters is critical to meet stringent quality standards and ensure the safety and efficacy of the final product. In the following, literature on the surface integrity in ECG have been reviewed.

Maksoud and Brooks [10] examined the ECG process on bronze samples using grinding wheels composed of diamond abrasive particles and metal bonds. Their study demonstrated that MRR improved by 30–40 % as the voltage and electrolyte flow rate increased. However, the grinding wheels wore up to 30 % faster. Bhuyan et al. [11] experimentally investigated MRR in ECG of aluminum alloy. They found that MRR increased from 0.03 mm³/min to 0.13 mm³/min as the applied current rose from 0.18 to 0.48 A, while other parameters remained constant, showcasing the strong influence of current on process efficiency. Hansong et al. [12] investigated material removal in ECG of turbine blades made of GH4169 alloy using special tools with internal electrolyte flushing system. The capabilities of cutting tools in surface machining have been studied. The results showed that a tool with conical shaped end improved the flatness. Increasing the feed rate up to 2.6 mm/min also improved the visual form of flat bottom surface, but it deteriorated with a rise in the voltage (10–25 V). Lyubimov et al. [13] studied the wear mechanisms of synthetic diamonds in ECG tools under grinding of a metal-ceramic hard alloys of WC–Co. the optimum. Their research identified optimal ECG parameters (voltage 5–8 V, current density 20–60 A/cm^2^, grinding force 1.5–2.0 MPa, grinding speed 12–15 m/s) in term of tool wear. In their optimum condition, the abrasive component of wear reached 80–85 % and the chemical one 15–20 %. Increase of the working voltage, grinding force and speed led to a raising of the chemical components up to 50 % and the total wear. Yehia et al. [14] investigated the effect of adding Al_2_O_3_ powder on MRR and surface roughness in the ECG of K110 alloy steel. The results showed an increase in MRR by increasing the Al_2_O_3_ just up to 5 wt%. The best MRR was observed at depth of cut 0 mm, v = 9V, and f = 6.81 in/min, with an improvement of 40.8 % in presence of Al_2_O_3_. The surface roughness was improved up to 37.66 % by increasing the feed rate and depth of cut, while decreasing the electrolyte concentration. Levinger and Malkin [15] performed ECG on tungsten carbide-cobalt (WC-Co) workpieces. Their experimental results estimated that the mechanical power required per unit volume of material removed during ECG could be a small fraction of the power needed for conventional grinding. Puri and Banerjee [16] optimized ECG parameters for grinding Tungsten carbide-P20 grade inserts. They showed voltage and cutting speed to be the two important parameters affecting the ECG, except for surface finish where only voltage is the predominant factor. The roughness analysis also showed the superiority of mechanical contribution to material removal at lower voltage and high cutting speed. the optimum Ra ≤0.8 μm was obtained at a voltage nearly 12 V. Roy et al. [17] also analyzed the effect of voltage on surface roughness in ECG of Tungsten carbide-P20 grade inserts and introduced a non-dimensional parameter as ‘periodicity to randomness index’. to find out the relative contribution of the electrolytic dissolution under the chosen experimental conditions. Li et al. [18] studied ultrasonic-assisted ECG (UAECG) on Ti-6Al-4V workpieces. The results showed a reduction in normal and tangential forces in UAECG by approximately 57 % and 56 %, respectively. Chip adhesion and grain fracture affected wheel life in ECG and UAECG, whereas wheel wear in conventional grinding was attributed to grain drop-off. Kozak and Skrabalak [19] analyzed abrasive-assisted ECG. They found that at an inter-electrode voltage of 6 V, mechanical grinding accounted for 70 % of material removal. However, at 10 V, electrochemical dissolution became dominant, contributing over 80 % of the total material removal. Sapre et al. [20] analyzed the effects of electrolyte flow in micro-ECG. To enhance the application of micro-ECG, a comprehensive study was conducted on the role of electrolyte flow in forming the oxide layer on the workpiece due to electrochemical dissolution and its removal by grinding wheel abrasion and electrolyte flow erosion. They found the electrochemical process contributed 70 % to total MRR, with grinding abrasion and electrolyte erosion contributing 20 % and 10 %, respectively. Electrolyte flow effects reduced oxide layer formation, improving machining accuracy. Lin et al. [21] conducted a study to improve the machined surface quality in ECG through optimum flushing of the electrolyte. In this research, the movement of machining debris under various flushing conditions was examined using numerical flow field simulations. The results revealed that debris removal speed significantly improved at a flushing pressure of 0.8 MPa and an electrolyte jet angle of 30°. Experimental results confirmed that the machined surface exhibited a metallic luster, clear edges, and grinding marks. Sonia [22] studied the ECG of mild steel and copper bar and compared the results versus conventional grinding. The results obtained by ECG revealed that the type of electrolyte and their concentration significantly affect the electrolysis in ECG. The 50 % reduction in roughness was found in ECG. Hu et al. [23], combined ECG with plunge internal grinding for finishing of bearing raceway. They showed Ra = 0.432 μm under the optimum condition (v = 10 V, f = 0.1 mm/min, and n = 6000 rpm. They also studied the surface residual stress under different conditions of electrolysis and demonstrated the reduction in compressive residual stresses due to electrochemical reactions and reduced portion of mechanical work. Zaborski et al. [24] stated the wear mechanisms of grinding wheel in ECG for G20 and titanium alloy WT3-1 and obtained wear of conventional grinding up to 8-times larger than ECG for both materials. They also demonstrated that using diamond embankment as grinding wheel decreased the relative wear up to 15 times smaller than conventional grinding. Gao et al. [25] proposed a novel Electrochemical Cleaning Grinding (ECCG) process to overcome the wheel loading issue in grinding of soft metals. The electrochemical process was applied to remove the adherent metal debris on the wheel surface during the grinding and maintain the grinding wheel sharpness. The tests were conducted on AA6061 and showed up to 70.1 % reduction in wheel loading and 51.5 % in roughness Ra compared to conventional grinding,

Despite the advancements in ECG as demonstrated in the literature, several research gaps remain. In the research conducted so far on ECG, the focus has primarily been on improving the process efficiency and increasing the MRR. However, a comprehensive investigation into the impact of process parameters such as voltage, current, cutting speed, and the electrolyte on surface integrity has not been studied. Most of the studies have addressed only one aspect of surface integrity, with fewer investigations considering these parameters simultaneously and their interactions. In addition, while studies have explored ECG for various materials and applications, there is a lack of focused research on the application of ECG specifically for AISI 304 grinding used in medical applications. The unique requirements for surface integrity in medical applications necessitate a tailored investigation and process optimization. Addressing these research gaps will provide a comprehensive understanding of the ECG process for AISI 304 stainless steel. This will enhance the manufacturing processes for medical devices, ensuring higher quality, better patient outcomes, and more efficient production methodologies. In this research, the effect of ECG process parameters on the surface integrity of AISI 304 samples was examined. To conduct this study, a customized ECG machine was designed, allowing precise control over a wide range of process parameters such as voltage, current, grinding wheel speed, and electrolyte concentration. Thereafter, surface integrity parameters including roughness, hardness, and texture were examined on ground samples. The main objective of this study was to identify and understand the effects of key process parameters on surface characteristics and to optimize the process conditions to achieve the best possible surface quality. The results of this research can contribute to improving the final quality of steel products and increasing the efficiency of the ECG process in various industries.

## 2 Material and Methods

**Fig. 1:**
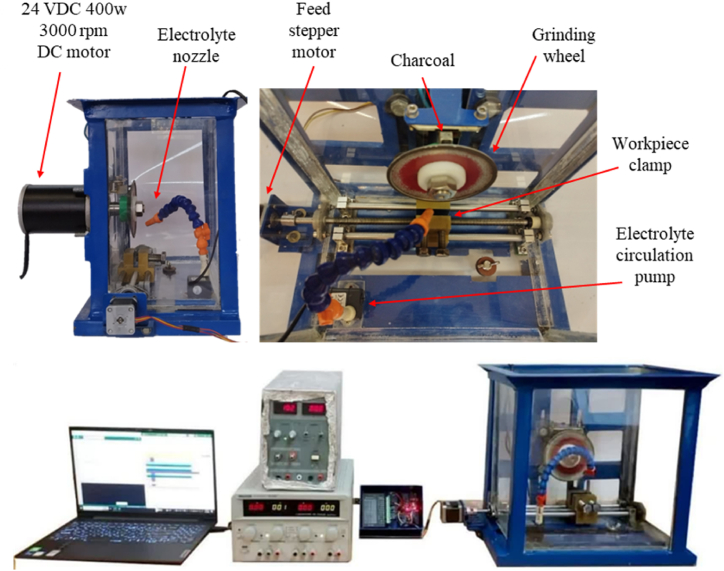
The ECG test setup used in experimental tests.

This study aims to study the effects of main process parameters on surface integrity in ECG of AISI 304. Accordingly, a custom-designed laboratory-scale ECG setup was developed with the capability to adjust the main process parameters (Fig. 1). The test setup consists of spindle drive and grinding wheel, electrolyte feed system, one dimensional feed control, and DC power supply. A contact brush contactor was used to transfer the electrical current to the rotating grinding wheel.

**Fig. 2:**
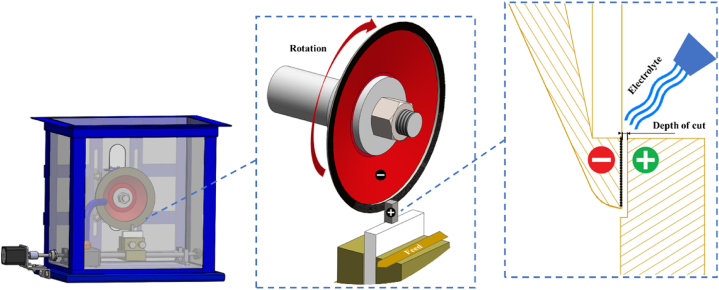
Schematic of the interaction between grinding wheel, electrolyte, and workpiece in ECG test setup.

Fig. 2 depicts the mechanism of ECG and interaction of wheel, workpiece, and electrolyte. The electrolytic cell is formed by applying polarity to the grinding wheel (cathode) and the workpiece surface (anode), with the electrolyte acting as a conducting liquid in between. This results in reduction at the cathode and oxidation at the anode. The grinding wheel plays a dual role in ECG: it mechanically removes material while also acting as a conductor for the electric current. A 120 mm diameter, cup-shaped grinding wheel Tyrolit was used which has a top layer of abrasive particles Al_2_O_3_ with a grit size of 125 embedded on the wheel surface using the metallic bond. The wheel is composed of both conductive and non-conductive materials, with the conductive component accounting for approximately 90 wt% of the wheel, while the abrasive particles account for only 10 %. The electrolyte was saline solution (mixture of distilled water and NaCl), flushing between the grinding wheel (cation) and the workpiece (anion) through a 5 mm nozzle. The electrolyte flow system was powered by a SUBO WP-3200 pump with a nominal flow rate of 5 L/min to ensure continuous electrochemical reactions. The motion of the dissolved ions causes the flow of electricity through the electrolyte and makes the solution electrically conductive. The conductivity of the electrolytes can be controlled by the electrolyte concentration. Anode dissolved product is removed by the electrolyte, which also dissipates the generated heat from the electrochemical reaction and abrasive grinding in the machining region.

**Fig. 3:**
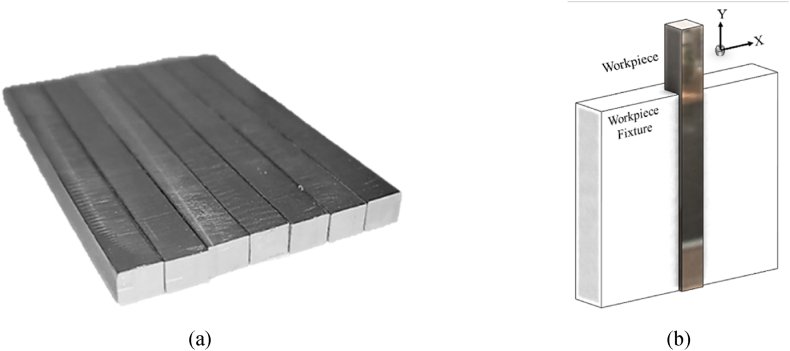
a) AISI 304 test samples used in experiments, b) workpiece clamping and insulation method.

Fig. 3 (a) shows the AISI 304 rectangular blocks of 6 × 6 × 60 mm with a hardness of 130 ± 3 Hv. The blocks were grounded, ultrasonically cleaned, and weighted by a precise balance before and after the ECG tests. The X and Y directions shown in Fig. 3 (b) represent the feed and perpendicular to feed directions, respectively. The samples were positioned and shielded from the machine clamp using a PTFE fixture.

To investigate the effect of ECG conditions on surface roughness, hardness, and surface texture when machining AISI 304 workpieces, a series of screening tests were conducted to determine the main process parameters and variation range. As stated before, this hybrid process dissolves the surface both electrochemically and mechanically. Except the electrochemical parameters, rotational speed, feed rate, abrasive grit size, and wheel composition, influence the balance between mechanical abrasion and electrochemical dissolution in ECG. The feed rate determines the contact time between the grinding wheel and the workpiece, with higher feed rates increasing the dominance of mechanical abrasion. Similarly, the abrasive grit size and the proportion of abrasive material in the wheel composition also affect the extent of material removal through mechanical grinding. Larger grit sizes or higher abrasive content promote more aggressive material removal, leaving more prominent feed marks on the surface. Conversely, finer grit sizes and optimized abrasive proportions contribute to smoother surfaces by reducing the mechanical footprint, especially when electrochemical action is dominant. In this study, the feed rate and wheel composition were kept constant to isolate the effects of the studied parameters (electrolyte concentration, voltage, and rotational speed). However, their influence on mechanical work and surface integrity remains significant and could be a subject for future exploration. Accordingly, three parameters including electrolyte concentration (Conc.), electrochemical process voltage (U), and grinding wheel rotational speed (n) were varied at five levels. Other cutting parameters were kept constant. The machining conditions are listed in Table 1. A central composite response surface methodology (RSM) was used for experimental design. Totally, twenty experiments were conducted, and the results were statistically analyzed using Minitab 21.

**Table 1:** Main ECG parameters along with the variation levels.

Process parameters	Levels				
	−2	−1	0	1	2
Voltage (U) (V)	5	10	15	20	25
Electrolyte concentration (Conc.) (g/L)	20	60	100	140	180
Rotational speed of grinding wheel (n) (rpm)	500	1000	1500	2000	2500
Feed rate (mm/min)	10				
Electrolyte temperature (°C)	25				
Depth of cut (mm)	0.1				
Length of cut (mm)	6				

The surface roughness (Ra) was measured using a Mahr-Marsurf PS1 roughness meter. Each sample's roughness was measured using a 1.75 mm evaluating length in both the X-direction (along the feed) and the Y-direction (perpendicular to feed). This evaluation length was selected to avoiding the overestimation near the edges, where potential surface irregularities or boundary effects exist when the grinding wheel enters the workpiece. To minimize the localizing effect, the measurements were repeated at three distinct sites and the average value of Ra was reported. The surface texture was examined using a VEGA3 TESCAN scanning electron microscope (SEM). The surface and in-depth microhardness were also measured using a Buehler hardness tester. The measurements were taken under a load of 0.1 kg for 4 s.

## 3 Results and discussion

As previously stated, twenty tests were conducted. Throughout the studies, the current density (J) was also measured. Table 2 shows the measured surface integrity parameters including, roughness, in depth hardness as well as current density in each experiment. The output parameters were analyzed using analysis of variance (ANOVA). In the following, each of these outcomes will be analyzed individually. Error assessment was also employed to validate the data and ensure the reliability of the experimental results.

**Table 2:** Experiments design matrix with output parameters.

No.	Conc. (g/L)	U (V)	n (rpm)	J (A/cm^2^)	Ra_y (μm)	Ra_x (μm)	H (Hv)			
							Surface	x20 μm	x 40 μm	x 60 μm
1	20	15	1500	200.3	0.848	0.709	138	138	133	134
2	60	10	1000	310.8	0.693	0.690	134	133	132	133
3	60	10	2000	138.7	0.656	0.696	145	138	134	133
4	60	20	1000	457.8	0.616	0.473	126	132	134	133
5	60	20	2000	374.5	0.609	0.473	134	136	132	132
6	100	5	1500	155.5	0.680	0.658	137	137	131	130
7	100	15	500	549.8	0.588	0.524	129	128	132	132
8	100	15	1500	452.2	0.423	0.384	134	133	132	130
9	100	15	1500	446.1	0.416	0.382	134	132	134	132
10	100	15	1500	450.2	0.412	0.382	133	133	133	133
11	100	15	1500	448.0	0.434	0.383	132	129	130	132
12	100	15	1500	442.3	0.449	0.381	134	133	132	132
13	100	15	1500	448.1	0.439	0.382	132	131	132	131
14	100	15	2500	305.3	0.402	0.535	142	136	133	133
15	100	25	1500	727.6	0.523	0.462	118	125	130	132
16	140	10	1000	448.7	0.473	0.413	134	133	132	133
17	140	10	2000	287.3	0.345	0.419	135	134	133	131
18	140	20	1000	813.5	0.421	0.434	121	122	131	130
19	140	20	2000	705.5	0.306	0.440	127	128	132	132
20	180	15	1500	683.4	0.352	0.392	124	127	131	130

### 3.1 Current density

The results indicate the variations of current density (J) in the range of 138.7–813.5 A/cm^2^. Table 3 shows the ANOVA of the current density after removing insignificant parameters. The study used a 95 % confidence level (P-value<0.05) to determine the significant parameters. The R-sq values for the current density model also represents the coverage of the output parameter by the model. The analysis indicated superior contribution of voltage and electrolyte concentration with the contributions of 50.8 % and 35.8 %, respectively with other parameters covering a contribution of lower than 10 %.

**Table 3:** ANOVA of current density (J) in ECG.

Source	DF	Adj SS	F-Value	P-Value	Contribution (%)
Conc.	1	235085	6818.1	<0.001	35.8
U	1	333514	9672.7	<0.001	50.8
n	1	64240	1863.1	<0.001	9.8
n^2^	1	523	15.2	0.002	0.1
Conc. × U	1	20022	580.7	<0.001	3.1
U × n	1	2527	73.3	<0.001	0.4
Error	13	448			0.1
Total	19	656359			100
R^2^ = 99.93 %, R^2^_adj._ = 99.90 %, R^2^_pre._ = 92.88 %					

The final model's R-squared value is 99.9 %, indicating that all the variance in current density can be explained by the model. Based on the developed model, the empirical formula (5) was obtained to estimate the current density.

(1) $$J=395.4-0.722\text{Conc}.-6.80U-0.1807n-0.000018n^{2}+0.2501\;\text{Conc}.\times U+0.007109\;U\times n$$

**Fig. 4:**
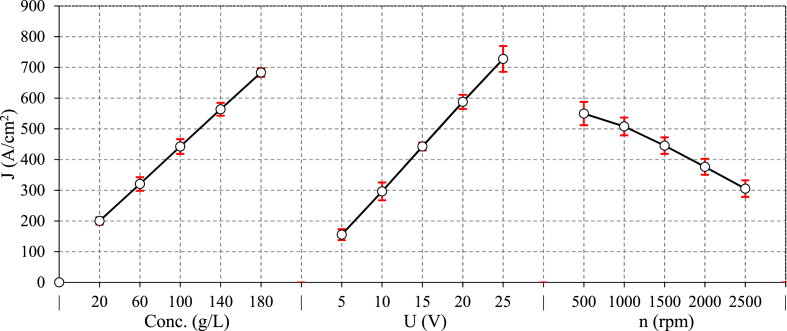
The main effect of ECG parameters on current density.

The effects of main ECG parameters including; electrolyte concentration, voltage, and rotational speed of grinding wheel on the current density are depicted in Fig. 4. As shown, the current density increases linearly with the electrolyte concentration and voltage. In fact, Higher electrolyte concentration enhances the conductivity of the electrolyte, allowing for more efficient current flow through the electrolyte as the number of dissolved ions in the water increases [26]. This improves electrochemical reactions and increased material dissolution, leading to a current density of up to 241 %. This also implies faster material removal at higher concentrations. On the other hand, voltage is the primary driving force behind the electrochemical reaction. As voltage rises from 5 V to 25 V, it accelerates the ionization process in the electrolyte, thereby enhancing the rate of electrochemical dissolution. This enhances rate of dissolution leads to a higher current density of up to 368 %. Ohm's law describes the linear relationship between voltage and current. A higher voltage facilitates the removal of material via electrochemical action, resulting in faster machining in ECG. However, increasing the rotating speed of the grinding wheel slightly decrease the current density up to 44.5 %. At higher rotary speeds, the contact period between the grinding wheel and the workpiece is shorter, resulting in a decrease in current density. This reduces the efficiency of the electrochemical process by allowing less time for ion exchange and dissolution. Furthermore, higher speeds may increase mechanical interaction (abrasion) in relation to electrochemical dissolution, limiting the contribution of electrochemical removal and decreasing current density. Furthermore, as the grinding wheel revolves at higher speeds, centrifugal forces push the electrolyte away from the contact zone between the grinding wheel and the workpiece. This reduces the availability of the electrolyte required for the electrochemical corrosion. The main effect plot of rotating speed also demonstrated any temperature increase due to higher rotational speeds appears to have a negligible effect on current density. This is largely because the cooling effect of the continuously flushing electrolyte helps maintain stable temperatures during the process.

**Fig. 5:**
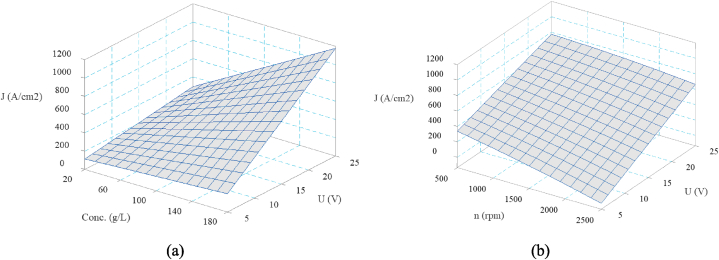
Interaction effect of ECG parameters on current density a) Conc. × U, b) n × U.

Fig. 5 depicts the impact of significant interactions on the current density. According to Fig. 5 (a), The maximum current density is attained at the maximum levels of electrolyte concentration and voltage. This is evidenced by the peak in the upper-right corner of the plot, where both the voltage and concentration are at their highest level. The rate of increase in current density becomes more prominent at higher values of both concentration and voltage, indicating that their combined effects are more significant than either alone. Fig. 5 (b) also illustrates the steeper relationship between electrolyte concentration and current density at lower rotational speeds.

### 3.2 Surface roughness

Surface roughness of the ground samples was measured at three regions and the average value was reported. Table 4 displays the ANOVA of the Ra model in feed (X) and perpendicular to the feed (Y) directions after removing insignificant parameters. ANOVA reveals the electrolyte concentration to be the main parameter affecting on the Ra, particularly in direction of perpendicular to the feed, with a contribution of 63.7 %.

**Table 4:** ANOVA of Ra model in both directions of X and Y in ECG.

Source	Ra_y					Ra_x				
	DF	Adj SS	F-Value	P-Value	Contribution (%)	DF	Adj SS	F-Value	P-Value	Contribution (%)
Conc.	1	0.255	816.0	<0.001	63.7	1	0.09924	40812.8	<0.001	33.6
U	1	0.017	55.9	<0.001	4.4	1	0.03901	16040.8	<0.001	13.2
n	1	0.027	86.8	<0.001	6.8	1	0.00010	41.1	<0.001	0.0
Conc.^2^	1	0.043	137.1	<0.001	10.7	1	0.04427	18203.7	<0.001	15.0
U^2^	1	0.044	139.6	<0.001	10.9	1	0.04944	20329.6	<0.001	16.8
n^2^	1	0.006	18.2	0.001	1.4	1	0.03390	13939.0	<0.001	11.5
Conc. × U	–	–	–	–	–	1	0.02904	11942.5	<0.001	9.8
Conc. × n	1	0.005	15.8	0.002	1.2	–	–	–	–	–
Error	12	0.004			0.9	12	0.00003			0.0
Total	19	0.401			100	19	0.29502			100
R^2^ = 99.01 % R^2^_adj._ = 98.44 % R^2^_pre._ = 95.77 %						R^2^ = 99.99 % R^2^_adj._ = 99.98 % R^2^_pre._ = 99.96 %				

Accordingly, the empirical model to estimate Ra surface roughness in Y and X directions, are presented in Eqs. (2), (3), respectively.

(2) $$\text{Ra}\_y=1.5463-0.006454\text{Conc}.-0.05662U-0.000139n+0.000026\text{Conc}.^{2}+0.001667U^{2}+0.0000001n^{2}-0.000001\text{Conc}.\times n$$

(3) $$\text{Ra}\_x=2.16334-0.011733\text{Conc}.-0.093210U-0.000436n+0.000026\text{Conc}.^{2}+0.001774\;U^{2}+0.0000001\;n^{2}+0.000301\text{Conc}.\times U$$

**Fig. 6:**
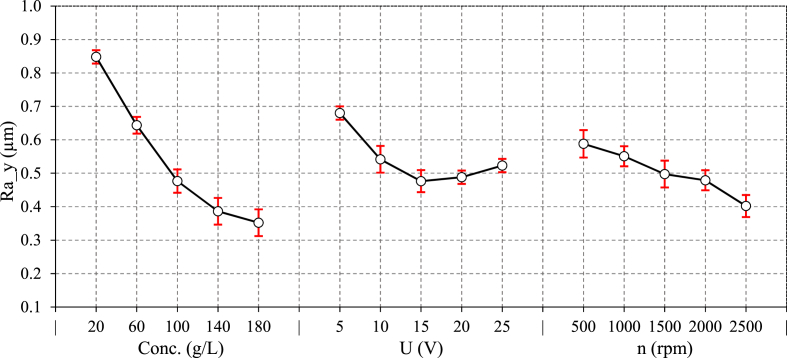
The main effect of ECG parameters on Ra along Y-direction.

**Fig. 7:**
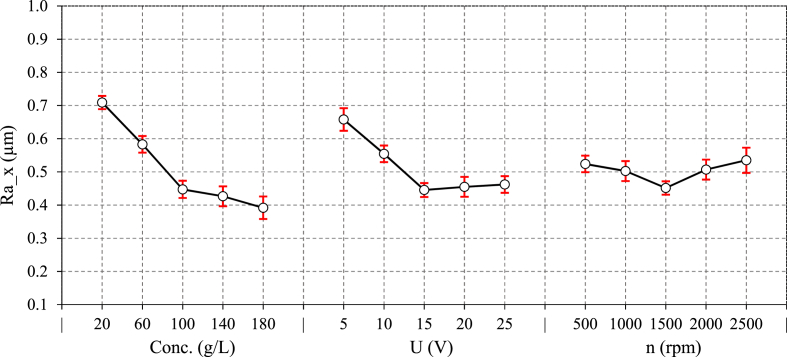
The main effect of ECG parameters on Ra along X-direction.

Fig. 6, Fig. 7 depict the variations of ECG major parameters on Ra in both directions. The results show that the process parameters have a lower effect on surface roughness in the parallel direction than in the perpendicular direction. This observation can be attributed to the fact that mechanical grinding feed lines mostly affect the roughness in the Y-direction. The material is removed not only by mechanical abrasion but also by electrochemical corrosion in ECG, which tends to eliminate the feed lines more effectively, thereby reducing the roughness in the Y-direction more efficiency. In fact, surface asperities may possess higher surface energy, which can influence the adsorption of corrosive species and corroded more rapidly [27]. Accordingly, increasing electrolyte concentration from 20 to 180 g/L, reduces the Ra by approximately 65 % and 45 % in the X and Y-directions, respectively. Increasing the electrolyte concentration in NaCl solution can increase the presence of chloride ions (Cl⁻), which are more aggressive toward metals. Higher NaCl concentrations increases the solution conductivity, which accelerates corrosion. Pitting corrosion is a prevalent type of corrosion in NaCl solution occurring when the surface oxide layer on the metal surface is damaged, particularly in stainless steels. Chloride ions are small passing through microscopic flaws in the oxide layer. Once the protective layer is breached, a localized anodic site (pit) forms, allowing metal dissolution occurs while the surrounding surface remains a cathode. This creates and aggressive, self-sustaining cycle in which chloride ions continue to attack the exposed metal [28,29]. Lower concentrations cause more localized dissolution, resulting in uneven surface corrosion. As electrolyte concentration increases, the uniform distribution of chloride ions results in more consistent corrosion across the entire surface, minimizing excessive pit formation. The corrosion uniformity smooths the surface and reduces roughness, particularly in the areas that might otherwise be affected by uneven material removal at lower concentrations. The variation of surface roughness (Ra) versus voltage in ECG shows a twofold effect due to the complex interactions between electrochemical dissolution and mechanical grinding. Surface roughness decreases by approximately 30 % up to 15 V, the slightly increases as voltage rises to 25 V. This pattern can be explained by the dual roles that voltage plays in the ECG process. Initially, increasing voltage directly boosts current density, leading to a higher MRR and more effective electrochemical dissolution. At lower voltages, the process is primarily governed by mechanical abrasion. Here, Abrasive particle size and wheel type have influence surface quality. However, as voltage rises and the electrochemical action takes over, aggressive dissolution helps smooth out the surface, reducing Ra by efficiently removing material and reducing the prominence of surface defects. The reduction in Ra at moderate voltages can also be attributed to the uniform increase in chloride anions produced during the electrochemical reaction. These anions attack the oxide layer, leading to more uniform material removal, especially in areas where the distance between the anode (workpiece) and cathode (grinding wheel) is close to the critical limit for effective electrochemical reaction. This uniform material removal leads to a smoother surface. However, beyond 15 V, the roughness increases slightly. This is due to over-etching and causes more aggressive material removal, leading to uneven corrosion or severe pitting on the surface. At higher voltages, localized spots may form due to non-uniform current distribution, especially in areas with surface roughness or irregularities. Thus, while increasing the voltage initially improves surface quality, excessive voltage can worsen it, emphasizing the importance of voltage control in ECG.

In the analysis of how wheel rotational speed affects Ra in ECG, two distinct trends emerge. In the Y-direction, increasing the rotational speed causes a 30 % decrease in Ra. As the grinding wheel rotates faster MRR becomes more efficient and decreases the size of grinding feed marks. In fact, the grinding process removes the oxidized layer from the workpiece surface, exposing a fresh metal to the electrolyte for oxidation. The oxidized layer is removed using mechanical grinding. As the wheel rotational speed rises, the oxidized layer is removed faster, resulting in lower roughness. However, Ra_x increases as the rotational speeds rises beyond 1500 rpm. In fact, Centrifugal forces may have pushed the electrolyte away from the grinding wheel-workpiece contact zone. As a result, the electrolyte is less effectively maintained at the interface, leading to a drop in current density. This is consistent with the current density results (Fig. 4). When the current density falls, the electrochemical dissolution becomes less effective, and mechanical effects dominate, explaining the increase in Ra.

**Fig. 8:**
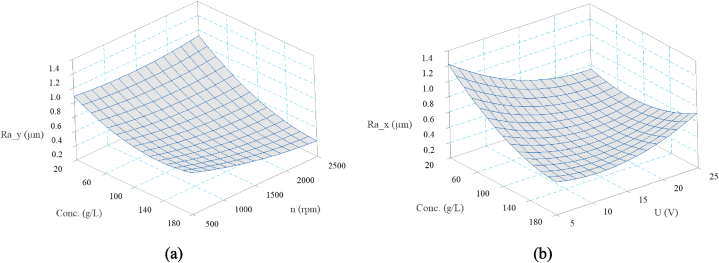
Interaction effect of ECG parameters on Ra along a) Y-direction, b) X-direction.

Fig. 8 shows the effects of significant interactions on Ra along both directions. Fig. 8 (a) illustrates that the decreasing effect of rotational speed is limited to higher electrolyte concentrations, indicating that removing oxidized layer is pronounced. Fig. 8 (b) also indicated the dual behavior of electrolyte concentration and voltage especially in X-direction. The maximum Ra is observed at both minimum and maximum interaction levels of theses parameters.

### 3.3 Surface microhardness

The microhardness was measured at both the surface and in-depth to study the effect of microstructure changes on the specimens. The ANOVA for surface microhardness is displayed in Table 5. Unlike the surface roughness, voltage is the main parameter affecting surface hardness with a 47 % contribution.

**Table 5:** ANOVA of surface hardness model in ECG.

Source	DF	Adj SS	F-Value	P-Value	Contribution (%)
Conc.	1	156.3	113.5	<0.001	19.4
U	1	380.3	276.2	<0.001	47.2
n	1	169.0	122.7	<0.001	21.0
Conc.^2^	1	6.9	5.0	0.045	0.9
U^2^	1	49.1	35.7	<0.001	6.1
n^2^	1	9.1	6.6	0.024	1.1
Conc. × n	1	18.0	13.1	0.004	2.2
Error	12	16.5			2.1
Total	19	805.1			100
R^2^ = 97.97 %, R^2^_adj._ = 96.79 %, R^2^_pre._ = 94.35 %					

Eq. (4) shows the formula for determining surface hardness based on ANOVA analysis.

(4) $$H=124.22+0.0997\text{Conc}.+0.702U+0.00677n-0.000327\;\text{Conc}.^{2}-0.05591U^{2}+0.000002\;n^{2}-0.000075\;\text{Conc}.\times n$$

**Fig. 9:**
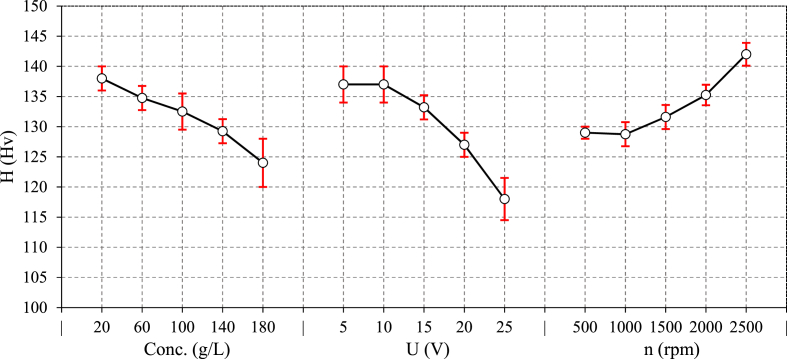
The main effect of ECG parameters on surface microhardness.

The effect of process parameters on microhardness, are depicted in Fig. 9. As the electrolyte concentration increases from 20 to 180 g/L, the surface hardness decreases by 10 %–124 Hv. It means the electrolyte concentration cause the surface softening even below the base hardness (130 ± 3 Hv). The hardness reduction is primarily due to surface corrosion. When the electrolyte concentration increases, so does the rate of electrochemical dissolution, causing more material being removed through corrosion. As a result, the surface resistant and hardness decrease. This phenomenon is also related to grain boundaries being attacked during the corrosion process, especially for alloys with varying chemical stability. The higher dissolution rate reduces hardness by preventing significant work hardening or plastic deformation. The effect of voltage on microhardness shows a similar trend, where increasing voltage decreases hardness up to 118 Hv, representing a 14 % decrease. At lower voltages, the electrochemical dissolution is not as aggressive, allowing the material to retain the hardness. However, the corrosion effect intensifies by increasing voltage, resulting in more material removal and surface softening. Over-etching or excessive dissolution can also lead to localized weakening of the surface structure. The effect of rotational speed on hardness exhibits a nonlinear trend. Initially, hardness is relatively constant but increases up to 142 Hv at higher levels of rotational speeds. In fact, mechanical forces may dominate electrochemical effects at higher speeds. In such cases, the MRR is higher due to the mechanical action, which may result in mild surface hardening [30]. Higher rotational speeds also reduce the workpiece exposure time to the electrolyte, leading to less corrosion and thus a higher retention of hardness. At high speeds, centrifugal forces may prevent an adequate amount of electrolyte from reaching the grinding interface, reducing the electrochemical dissolution and increase in hardness.

**Fig. 10:**
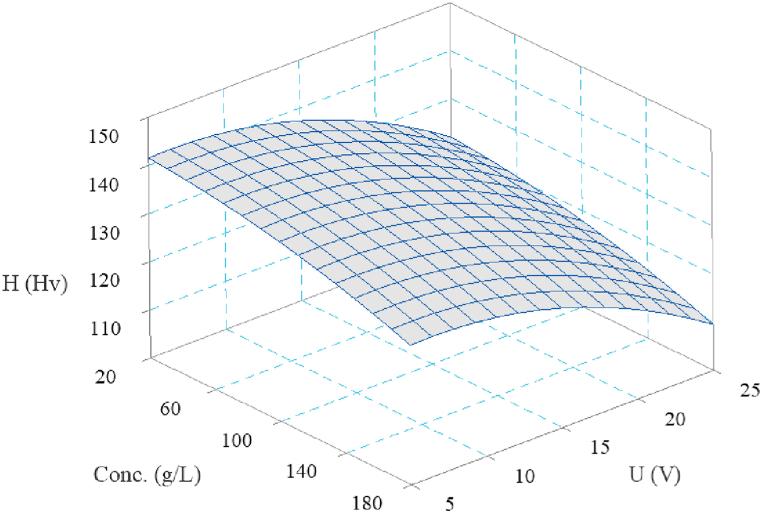
Interaction effect of ECG parameters on surface microhardness.

Fig. 10 shows a surface plot illustrating the combined effects of electrolyte concentration and voltage on the surface microhardness. Accordingly, the hardness is higher at low concentrations and voltages, indicating less aggressive material removal and lower corrosion rates. However, as either the concentration or voltage increases, the hardness drops, suggesting that both parameters contribute to the material softening. At extremely high concentrations and voltages, the effects of further increasing these parameters seem to plateau, indicating that the system may have reached a state of excessive dissolution, in which the electrochemical reaction rapidly removes material while not causing additional softening.

**Fig. 11:**
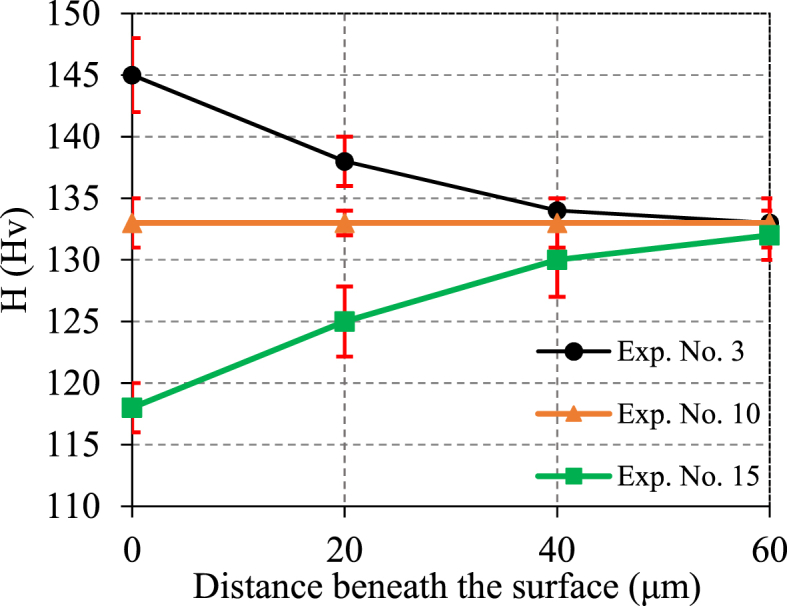
Distribution of in-depth microhardness in ECG at experiments No. 3, 10, and 15.

Fig. 11 illustrates the variation of in-depth microhardness in experiments No. 3, 10, and 15. Experiments No. 3 (Conc. = −1, U = −1, n = 1) showed the maximum surface hardness of 145 Hv. As mentioned before, the increase in hardness at higher rotational speed is a result of more portion of mechanical work compared to other experiments. On the other hand, experiments No. 15 (Conc. = 0, U = 2, n = 0) shows a slight drop in surface hardness up to depth of 30 μm compared to base hardness (130 ± 3 Hv) due to excessive corrosion at highest level of voltage. Regarding the microhardness results for Experiment No. 10, the consistent hardness observed across all depths can be explained by the stable conditions of the ECG process. The process parameters in this experiment were set at levels that maintained a balance between electrochemical dissolution and mechanical abrasion. As a result, the process neither introduced significant work hardening nor caused excessive corrosion, keeping the microhardness steady and close to the base hardness of the material.

### 3.4 Surface texture

**Fig. 12:**
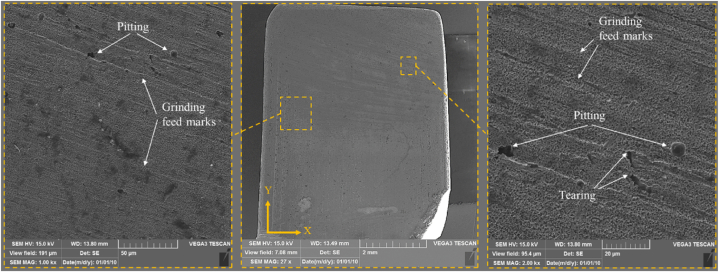
SEM images of samples after ECG in experiment No. 3 (Conc. = 60 g/L, U = 10 V, n = 2000 rpm).

The final surfaces were examined using SEM to study the surface alteration. Fig. 12 illustrates the surface morphology following an ECG in experiment No. 3 (Conc. = −1, U = −1, n = 1). The presence of grinding feed marks and tearing in all images suggests that the mechanical grinding in dominant over corrosion at higher rotation speeds. In ECG, when the electrochemical dissolution is insufficient due to lower concentration and lower voltage, the mechanical abrasion of the grinding wheel contributes more to material removal. This is why grinding feed marks are clearly visible, as there is insufficient corrosion to smooth out mechanical scratches. The corrosion is also visible as pitting, albeit less pronounced.

**Fig. 13:**
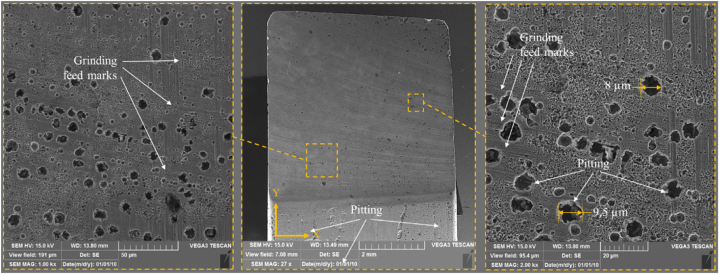
SEM images of samples after ECG in experiment No. 2 (Conc. = 60 g/L, U = 10 V, n = 1000 rpm).

Fig. 13 shows the surface texture following ECG in experiment No. 2 (Conc. = −1, U = −1, n = −1). In comparison to Fig. 12, decreasing the rotational speed improves the corrosion rate of the surface during ECG while also reducing the portion of mechanical work, as seen by the more prominent pitting and reduced feed marks. Pitting is caused by a combination of electrochemical reactions, material composition, and mechanical factors. The low rotational speed allows chloride-rich electrolyte solution to enter these cavities. Chloride ions are extremely corrosive and significantly reduce the material's corrosion resistance by initiating and accelerating the anodic dissolution process within the cavities. These chloride ions not only initiate corrosion but also maintain it, creating a continuous cycle in which pits deepen and widen. Once the chloride ions enter the pits, they facilitate both anodic and cathodic half-reactions in the same localized area. The anodic reaction dissolves metal, whereas the cathodic reaction consumes oxygen. The lack of oxygen inside these cavities causes a significant drop in corrosion resistance, making these areas highly susceptible to further dissolution. As a result, the pits expand in both depth and diameter, leading to large cavities of up to ten μm (experiment No. 3).

**Fig. 14:**
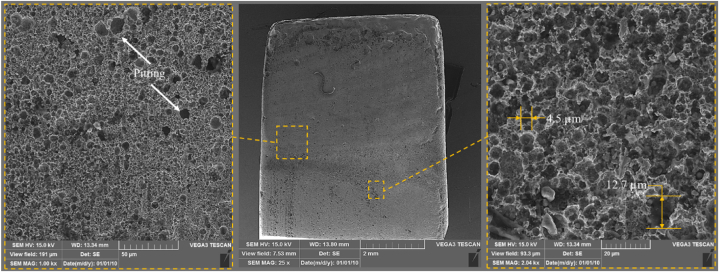
SEM images of samples after ECG in experiment No. 17 (Conc. = 140 g/L, U = 10 V, n = 2000 rpm).

The surface texture of the experiment No. 17 (Conc. = 1, U = −1, n = 1) is shown in Fig. 14. The image of pitting across the entire workpiece surface suggests a dominant electrochemical effect caused by a higher electrolyte concentration. The higher ionic concentration causes more aggressive and uniform material dissolution, resulting in pitting across the surface. However, the presence of chloride ions becomes more dominant as the concentration increases. These ions penetrate the surface and enter the grain boundaries, causing the anodic and cathodic half-reactions within the pits. The chloride ions widen the pits by breaking down the passive oxide layer. However, the roughness measurements shows that the pits are shallow, which improves the final surface roughness.

**Fig. 15:**
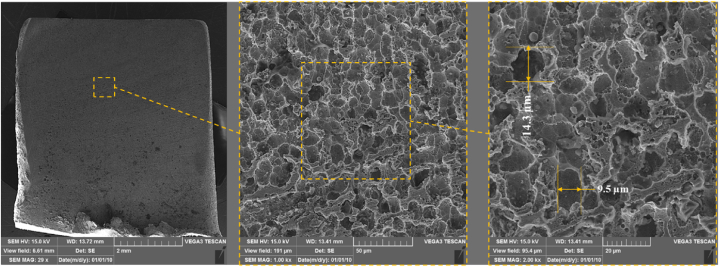
SEM images of samples after ECG in experiment No. 18 (Conc. = 140 g/L, U = 20 V, n = 1000 rpm).

Fig. 15 shows the surface texture after ECG in experiment No. 18 (Conc. = 1, U = 1, n = −1). The SEM image showing more uniform pitting across the entire surface, indicates that the combination of higher electrolyte concentration, increased voltage, and lower rotational speed leads to a more consistent electrochemical reaction. The higher voltage amplifies the current density, further accelerating the material dissolution. However, increasing the voltage to an optimum level ensures that the corrosion affects the entire surface uniformly. In fact, when compared to Fig. 14, the combination of higher voltage, and lower speed produces a slightly smoother, more evenly corroded surface, characterized by a uniform distribution of pits rather than localized or sporadic corrosion.

## 4 Conclusion

This study investigated the influence of main process parameters in ECG of AISI 304, focusing on key outcomes including current density, surface roughness, microhardness, and surface texture using a customized test setup. The main results are highlighted as following:

• Voltage and electrolyte concentration emerged as the dominant factors increasing the density, up to 368 % and 241 %, respectively in the studied range. Higher voltage accelerated ionization and dissolution, while increased concentration enhanced electrolyte conductivity. In addition, high rotational speeds pushed the electrolyte away from the cutting zone due to centrifugal forces and decreased the current density.
• Surface roughness significantly decreased as the electrolyte concentration and voltage increased, particularly in the Y-direction (perpendicular to feed) up to 65 %. dilute solution and overvoltage resulted in uneven material removal by corrosion mechanism and increasing the surface roughness.
• Higher electrolyte concentration and voltage caused a 10 % and 14 % drop in surface hardness, respectively, due to intensified corrosion (lower than material initial hardness). However, at extremely high rotational speeds, hardness increased up to 142 Hv as mechanical forces began to dominate over electrochemical effects.
• SEM images of surface texture revealed more uniform pitting across the surface at higher electrolyte concentrations and voltages, especially at lower rotational speeds. This uniform material dissolution demonstrated that these conditions favored more consistent electrochemical reactions, leading to smoother and more evenly corroded surfaces. However, higher voltage leads to over-etching and formation of deep pitting which results in higher roughness.

## CRediT authorship contribution statement

**Mohammad Yazdani:** Writing – original draft, Visualization, Validation, Investigation, Formal analysis. **Amir Rasti:** Writing – review & editing, Supervision, Project administration, Methodology, Formal analysis, Data curation, Conceptualization.

## Data availability

Data will be made available on request.

## Declaration of Competing Interest

The authors declare that they have no known competing financial interests or personal relationships that could have appeared to influence the work reported in this paper.
